# Membrane-Anchored Serine Protease Inhibitors: Physiological Functions, Mechanisms, and Roles in Cancer

**DOI:** 10.3390/ijms27042000

**Published:** 2026-02-19

**Authors:** Chun-Ying Chen, Tai-No Lin, Hsiang-Po Huang

**Affiliations:** 1 School of Medicine, College of Medicine, National Cheng Kung University, Tainan 701401, Taiwan; i54081055@gs.ncku.edu.tw; 2 Graduate Institute of Medical Genomics and Proteomics, College of Medicine, National Taiwan University, Taipei 100233, Taiwan; d14455003@ntu.edu.tw

**Keywords:** HAI-1, HAI-2, type II transmembrane serine protease, matriptase, hepatocyte growth factor activator, epithelial homeostasis, cancer, epigenetic regulation

## Abstract

Pericellular proteolysis is essential for maintaining tissue homeostasis. Central to this process are hepatocyte growth factor activator inhibitor-1 (HAI-1) and HAI-2, membrane-bound inhibitors that regulate type II transmembrane serine proteases, including matriptase and prostasin, through high-affinity Kunitz domains. This review summarizes current understanding of their molecular structures, physiological roles, and cancer-related clinical relevance. Genetic models reveal HAI-1 is critical for placental and skin development, while HAI-2 is crucial for neural tube closure and intestinal integrity. In cancer, HAIs generally act as tumor suppressors. Their downregulation, often via promoter hypermethylation, leads to excessive activation of hepatocyte growth factor/c-MET or protease-activated receptor-2/NF-κB signaling, promoting epithelial–mesenchymal transition and cancer progression. Clinically, reduced HAI levels in tumors correlate with metastasis and poor prognosis in several carcinomas. Paradoxically, elevated HAI expression in certain cancers suggests context-dependent pro-tumor functions. Emerging evidence links HAI loss to immune suppression, notably via M2 macrophage polarization in lung cancer. Finally, we highlight future directions for identifying tissue-specific serine proteases, downstream signaling, and therapeutic strategies, including recombinant mimetics and epigenetic reactivation, in precision oncology. In conclusion, HAI-1 and HAI-2 are key regulators of tissue homeostasis and cancer, with overlapping yet distinct functions, which present promising opportunities for therapeutic targeting.

## 1. Introduction

Serine proteases are a versatile group of enzymes characterized by a serine residue in their active site, which is essential for their proteolytic activity. These enzymes participate in fundamental physiological processes such as digestion, blood coagulation, immune defense, and cell signaling [1]. Type II transmembrane serine proteases (TTSPs) are a subfamily of serine proteases widely distributed across vertebrates and some invertebrates. They are characterized by a cytoplasmic domain that potentially mediates intracellular signal transduction and an extracellular domain that catalyzes substrate cleavage [2]. TTSPs are involved in various biological processes including tissue development, epithelial integrity, host defense, and cellular signaling [3]. Several TTSP members, including matriptase, transmembrane protease, serine 2 (TMPRSS2), and hepsin, are also implicated in pathological states, such as cancer progression, making them important targets for therapeutic intervention [3].

To counterbalance the potent activity of cell-surface serine proteases, many tissues express membrane-anchored serine protease inhibitors. The two best-studied inhibitors of TTSPs are hepatocyte growth factor activator inhibitor-1 (HAI-1), encoded by the serine peptidase inhibitor, Kunitz Type 1 gene (*SPINT1*), and hepatocyte growth factor activator inhibitor-2 (HAI-2), encoded by *SPINT2* [4]. The significance of these inhibitors became evident with the discovery of HAI-1, a novel Kunitz-type inhibitor identified during purification of hepatocyte growth factor (HGF) activator (HGFA), a protease responsible for converting pro-HGF into its active form [5]. Shortly thereafter, HAI-2 was purified [6]. Subsequent studies revealed that HAI-2 shares a structural organization similar to HAI-1, featuring two extracellular Kunitz-type domains. Notably, the three-dimensional structures of the main extracellular regions of both HAI-1 [7,8] and HAI-2 [9] have been characterized by X-ray crystallography.

Beyond their biochemical identification, HAI-1 and HAI-2 have been shown to play indispensable roles in diverse physiological contexts. For example, HAI-1 has critical roles in skin barrier formation and epidermal differentiation [10]. In the gastrointestinal tract, HAI-1 is required for maintenance of intestinal epithelial integrity [11]. In reproduction and development, HAI-1 is indispensable for placental labyrinth morphogenesis [12]. Similarly, HAI-2 is required for embryonic development, including placentation and neural tube closure [13]. In the airway, HAI-1 expression is upregulated after tissue injury, supporting its role in epithelial repair [14], while HAI-2 inhibits TMPRSS2- and matriptase-dependent activation of influenza and metapneumoviruses, highlighting its contribution to airway defense [15]. Collectively, these findings establish HAI-1 and HAI-2 as versatile regulators of protease activity in both epithelial and non-epithelial contexts.

This review provides a broad overview of the physiological roles of HAI-1 and HAI-2 in normal development and highlights how their dysregulation in cancer reflects a pathological mirror of these processes. By linking basic biology with tumorigenesis, we underscore their relevance as key regulators in both health and cancer. Despite growing interest, an integrated view of HAI-1 and HAI-2 in both development and cancer remains lacking, making this synthesis both timely and necessary. We first summarize their molecular architectures, regulatory mechanisms, and subcellular localizations, followed by a discussion of their diverse physiological and developmental roles across multiple organ systems. We then highlight the clinical consequences of genetic mutations in *SPINT1* and *SPINT2*. The review further explores the dysregulation of HAI-1 and HAI-2 in cancer, where they influence epithelial–mesenchymal transition, tumor immune evasion, and key oncogenic pathways such as HGF/mesenchymal–epithelial transition factor (MET) and Wnt/β-catenin. Finally, we discuss therapeutic opportunities ranging from biomarker applications to epigenetic reactivation strategies.

## 2. Research Design and Characteristics

### 2.1. Databases and Search Strategies

We performed a comprehensive literature search of PubMed from their inception to 25 August 2025. Two investigators independently screened full-text articles for eligibility. The search strategy incorporated the following terms: (HAI-1) OR (Spint1) OR (Hepatocyte growth factor activator inhibitor-1) OR (Serine peptidase inhibitor, Kunitz type 1) OR (HAI-2) OR (Spint2) OR (Hepatocyte growth factor activator inhibitor-2) OR (Serine peptidase inhibitor, Kunitz type 2).

We also manually reviewed the reference lists of eligible studies to include or exclude any additional relevant publications.

### 2.2. Study Eligibility Criteria

- Inclusion Criteria

1. Original research articles or review articles focusing on HAI-1 or HAI-2.
2. Studies addressing cancer biology–related topics.

B. Exclusion Criteria

1. Articles without full-text availability or not written in English.
2. Studies in which HAI-1 or HAI-2 were addressed only minimally (e.g., 1–2 sentences in the Discussion section).
3. Non-cancer studies limited to clinical correlations of HAI-1 or HAI-2, without accompanying functional or mechanistic analyses, as such studies primarily address disease-associated contexts rather than clearly defining physiological roles.

A total of 171 studies were included, most of which investigating the molecular mechanisms, biological functions, regulatory pathways, or pathophysiological roles of HAI-1 and HAI-2, as well as their roles in different cancers.

## 3. Molecular Structure and Regulation

### 3.1. Domain Architecture

**Figure 1:**
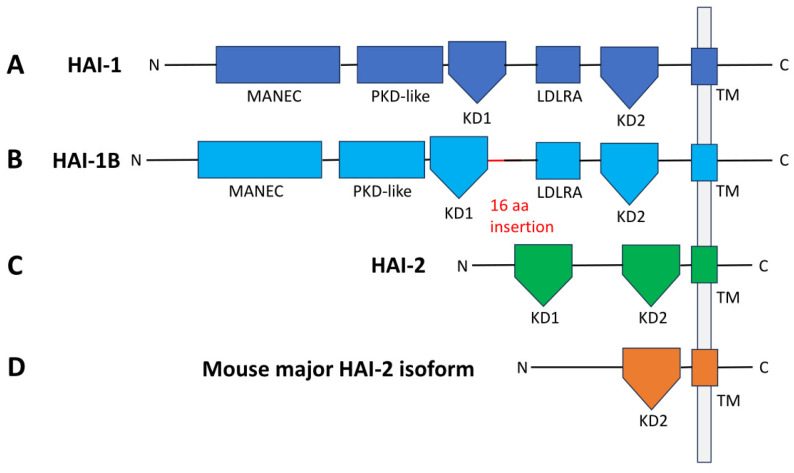
Domain architectures of HAI-1 and HAI-2 isoforms. (A) Human HAI-1: The HAI-1 protein comprises an N-terminal MANEC domain, an internal PKD-like domain, KD1, an LDLRA domain, and KD2, followed by a TM region and a cytoplasmic tail. (B) Human HAI-1B: Human HAI-1B is an alternative splice variant of HAI-1. The structure is identical to HAI-1 but contains an additional 16-amino-acid insertion immediately downstream of the KD1 domain. (C) Human HAI-2: HAI-2 is a shorter transmembrane protein lacking the MANEC and LDLRA domains found in HAI-1. (D) Mouse major HAI-2 isoform: Unlike human HAI-2, the predominant isoform in mice lacks the KD1 domain. This isoform contains only the KD2 domain in its extracellular region. Human and murine HAI-1 exhibit highly similar overall domain architectures. N: N-terminus, C: C-terminus, TM: transmembrane.

HAI-1 is a 529-amino-acid, 66 kDa multidomain protein [5,7], comprising an extracellular region (or a secreted 58 kDa variant) that includes, from the N terminus, a motif at N terminus with eight cysteines (MANEC) domain, an internal polycystic kidney disease (PKD)-like domain, Kunitz domain 1 (KD1), a low-density lipoprotein receptor class A (LDLRA) domain, and Kunitz domain 2 (KD2), followed by a single-span transmembrane region, and a short C-terminal cytoplasmic tail (Figure 1). HAI-1 contains three predicted N-glycosylation sites located at Asn-66, Asn-235, and Asn-523 [8]. Disulfide bonds are crucial for the structural integrity, stability, and inhibitory function of HAI-1 and HAI-2 proteins. HAI-1 has multiple cysteine-rich domains, including MANEC (with 8 cysteines forming 4 disulfides), two Kunitz domains (each with 6 cysteines forming 3 conserved disulfides), and LDLRA domain (6 cysteines possibly forming 3 canonical disulfide bonds [16]). The MANEC domain’s distinctive disulfide bonds enhance domain compaction and intramolecular interactions by anchoring the N terminus to α helix 2 and bridging strands 2 and 4 to project a loop outward, thereby defining a novel Plasminogen Apple Nematode subclass [17].

Early studies demonstrated that KD1 was the major inhibitory unit against hepatocyte growth factor activator (HGFA), while the KD2 showed negligible activity toward HGFA but retained inhibitory potency against trypsin [18]. Interestingly, the coexistence of both domains reduced the inhibitory efficacy of Kunitz I toward HGFA, suggesting steric interference between these two domains [18]. The importance of KD1 was further highlighted in glioblastoma models, where disruption of KD1 abolished anti-invasive effects, while KD2 contributed only marginally [19]. Alternative splicing was also shown to generate an isoform such as HAI-1B, which differs from canonical HAI-1 by an additional 16 amino acids located downstream of the KD1 domain (Figure 1), yet both variants rely on KD1 as their principal inhibitory unit [19,20].

Further dissection of domain interactions revealed that KD1 remained the principal inhibitory unit of HAI-1 against matriptase, but its potency was modulated by surrounding structural elements [21]. The N-terminal domain and KD2 were shown to attenuate KD1 activity by reducing the association rate, while the internal PKD-like domain enhanced both association rate and stability of the KD1–protease complex. The LDLRA domain also influenced binding kinetics by maintaining correct domain orientation [21]. These findings established HAI-1 as a flexible multidomain inhibitor, with auxiliary domains fine-tuning KD1 function. Nuclear magnetic resonance analysis of the MANEC domain further revealed it to be a novel subclass of the plasminogen–apple–nematode domain family, characterized by additional disulfide bonds, extended loops, and α-helical elements. This suggested that the MANEC domain was not merely a stabilizing motif but may actively mediate protein–protein interactions [17]. Crystallographic studies then demonstrated that the internal PKD-like domain folds and forms a compact V-shaped architecture with KD1 [7].

Integrative crystallographic and small-angle X-ray scattering analyses revealed that soluble HAI-1 adopts a compact conformation in which KD1′s reactive loop is occluded by neighboring domains and the 365-linker (residues 354–374), establishing an auto-inhibited state [8]. N-glycosylation at the MANEC domain further contributes to this masking. Small-angle X-ray scattering analysis of extracellular HAI-1 in complex with matriptase serine protease domain showed that only upon matriptase binding does HAI-1 shift into an extended conformation, exposing KD1 for inhibition. These findings established a novel auto-inhibitory mechanism governed by interdomain contacts and linker elements [8]. Regarding to the KD2 domain, KD2 of HAI-1 is functionally significant primarily for trypsin inhibition, but is largely inactive against HGFA and matriptase [18,21]. However, KD2 may play a modulatory role by influencing the overall conformation and regulatory properties of HAI-1 [21].

HAI-2 is a 252-amino-acid, ~26 kDa (unglycosylated) multidomain protein, comprising an extracellular region (or a 14 kDa secreted form) with KD1 and KD2, a transmembrane domain, and C-terminal cytoplasmic tail, but lacking MANEC or LDLRA domain (Figure 1) [6]. HAI-2 contains a KD1 domain similar to HAI-1, whereas their KD2 domains are more divergent. Among the two putative N-glycosylation sites, Asn-57 of HAI-2 is required for correct protein folding and protease inhibitory function [22]. Structural information on HAI-2 is limited; however, X-ray crystallography of the mesotrypsin–HAI-2 KD1 complex revealed a compact KD1 structure stabilized by three conserved disulfide bonds [9]. Although mouse HAI-2 encodes two Kunitz domains, the predominant isoform lacks KD1 (Figure 1); notably, its remaining KD2 domain efficiently inhibits HGFA, making it a potent in vivo inhibitor and highlighting species-specific regulation compared to human HAI-2 [23,24]. In addition, clinical genetic studies have identified missense mutations in KD2 of HAI-2 in patients with syndromic congenital sodium diarrhea (SCSD). These variants selectively impair inhibition of prostasin while preserving inhibition of matriptase, demonstrating that KD2 contributes to substrate selectivity rather than serving as a primary inhibitory unit [25].

### 3.2. Factors Affecting Binding and Inhibitory Activity

The binding and inhibitory properties of HAI-1 and HAI-2 toward their protease partners have been quantitatively characterized. For HAI-1, KD1 binds matriptase with high affinity (Kd = 13 ± 2 pM) [26], whereas KD2 shows no detectable binding. Functionally, extracellular HAI-1 and KD1 inhibit matriptase with Ki values of 9.1 ± 1.0 nM and 0.3 ± 0.02 nM, respectively [26]. KD1 also preferentially inhibits matriptase relative to hepsin (~1 nM), HGFA (3.9 nM), and plasma kallikrein (105 nM) [7]. In contrast, HAI-2 KD1 and KD2 exhibit comparable affinities for the matriptase protease domain, with Kd values of 35–45 nM [27]. Sequence features of the binding loop strongly influence protease recognition and inhibitory specificity. Selective inhibition of matriptase by HAI-1 is mediated by KD1 rather than KD2, and crystallographic analyses indicated that residues at the P1, P3, and P1′ positions are critical for discriminating between the two domains [28]. Additional specificity determinants include a phenylalanine at the P3′ position of HAI-1, which enhances the size of hydrophobic interactions, and a P3 arginine that engages the S3 pocket of matriptase rather than the S4 pocket, as observed in other serine protease-inhibitor cases [28]. Disulfide bonds stabilize reactive-loop conformations required for effective binding [28] and contribute to the unique structural features of the MANEC domain [8], which attenuates HAI-1′s inhibitory activity [21]. Beyond loop sequence, HAI-1 displays a unique KD1–KD2 arrangement in which both reactive sites are partially occluded by neighboring structural elements, providing an additional regulatory layer [8]. Finally, intramolecular electrostatic and hydrophobic interactions further modulate inhibitory-loop accessibility [8].

### 3.3. Subcellular Localization Differences Between HAI-1 and HAI-2

Despite their close structures, HAI-1 and HAI-2 exhibit strikingly different subcellular localization patterns that profoundly influence their inhibitory functions. HAI-1 is predominantly expressed on the cell surface, particularly the basolateral membrane of polarized epithelial cells. Detailed trafficking studies demonstrated that HAI-1 is first exocytosed to the basolateral membrane, underwent rapid endocytosis, and then recycled between endosomes and the plasma membrane before being partially transcytosed to the apical domain. This dynamic itinerary ensures that HAI-1 remains in close proximity to matriptase at sites of zymogen activation, allowing efficient and timely inhibition [29,30].

In contrast, HAI-2 is largely retained within intracellular compartments, including vesicle- or granule-like structures, due to incomplete plasma membrane export motifs and the presence of multiple Arg/Lys-rich endoplasmic reticulum (ER) retention-like sequences. As a result, HAI-2 often fails to access cell-surface matriptase or prostasin, rendering it less effective in controlling extracellular proteolysis [22,31]. For example, in mammary epithelial cells, HAI-2 remains intracellular during matriptase activation, and matriptase inhibition is exclusively mediated by HAI-1. However, in breast cancer cells, misrouting of HAI-2 to the plasma membrane allows it to interact with matriptase, forming stable inhibitory complexes and contributing to regulation of pericellular proteolysis [32]. Post-translational modifications also contribute to these localization differences. In Caco-2 cells (human colorectal adenocarcinoma-derived epithelial cell line), HAI-2 is synthesized in two glycoforms: an oligomannose-type species are retained in the ER/Golgi and a complex-type glycosylated form is targeted to apical vesicular structures [22]. Only the complex-type glycoform gains access to cell-surface proteases such as matriptase and prostasin. Mutation of the critical glycosylation site Asn57 disrupts N-glycosylation of both HAI-2 isoforms and causes improper folding and targeting, leading to mislocalization and loss of inhibitory activity [22].

### 3.4. Multi-Layered Regulation of HAI-1 and HAI-2

#### 3.4.1. Epigenetic Regulation

Both HAI-1 and HAI-2 genes are subjective to epigenetic control, with most evidence derived from cancer-related studies. Aberrant up-regulation of HAI-1, due to promoter hypomethylation, has been observed in hepatocellular carcinoma (HCC) and is associated with poorer tumor differentiation. Treatment with 5-aza can further increase HAI-1 expression [33].

The epigenetic regulation of HAI-2 was discovered even much earlier than that of HAI-1, most notably through CpG-island DNA methylation within its promoter region. In HCC tissues, *SPINT2*/placental bikunin showed dense promoter hypermethylation linked to transcriptional repression [34]. Future research showed that promoter hypermethylation of *SPINT2* was an important epigenetic event linking chronic hepatitis C virus infection to hepatocarcinogenesis, highlighting its potential as a biomarker for risk assessment and early detection of HCC [34,35]. Promoter hypermethylation also contributes to *SPINT2* silencing in gliomas, linking its loss to enhanced invasiveness and malignant transformation [36]. In high-grade gliomas, *SPINT2* promoter hypermethylation is frequent (33.3% of grade II, 71.4% of grades III, and 74.3% of grade IV), associated with reduced *SPINT2* messenger ribonucleic acid (mRNA) and accompanied by intact expression of target proteases such as HGF activator.

Forced expression of *SPINT2* reduces MET phosphorylation and suppresses glioblastoma growth in vitro and in intracranial xenografts in nude mice [37]. An integrated methylome–transcriptome analysis further showed that *SPINT2* is hypermethylated in both IDH1-mutant and wild-type glioblastomas. Moreover, demethylating agents or knockdown of DNA methyltransferase 1 (DNMT1) restore *SPINT2* expression, down-shift MET activation, and blunt malignant phenotypes of glioblastoma [38]. *SPINT2* also acts as a tumor suppressor gene in medulloblastoma. Its frequent epigenetic silencing, coupled with occasional genetic deletions, removes inhibition of the HGF/MET signaling pathway, thereby contributing to medulloblastoma pathogenesis [39]. Additionally, a functional epigenomics approach in renal cell carcinoma identified *SPINT2* as a methylation-inactivated tumor suppressor candidate that could be re-expressed by demethylating treatment [40]. In primary gastric cancers, hypermethylation is detected in 75% (30/40) of tumor tissues but is absent in adjacent normal mucosa. *SPINT2* mRNA expression is markedly reduced in gastric cancer cell lines; however, treatment with the 5-aza-2′-deoxycytidine (decitabine) restored its expression. Functionally, restoring *SPINT2* expression in gastric cancer cells inhibits proliferation, induces apoptosis, reduces anchorage-independent growth, and suppresses tumor formation in nude mice xenografts [41]. Consistent observations have been reported in esophageal squamous cell carcinoma, where *SPINT2* promoter methylation correlates with transcriptional down-regulation and loss of tumor-suppressive effect, which can be restored upon *SPINT2* re-expression [42]. Another cervical carcinoma study using methylation-specific polymerase chain reaction (PCR) and bisulfite sequencing confirmed promoter methylation of *SPINT2* in tumors and demonstrated its re-expression after 5-azacytidine treatment in cell models [43]. *SPINT2* downregulation in the bone marrow microenvironment, mediated by promoter methylation, facilitates aberrant cytokine secretion (e.g., HGF and CXCL12), extracellular matrix remodeling, and abnormal adhesion of leukemic stem cells, thereby supporting leukemia progression [44]. Restoration of *SPINT2* expression by demethylating therapy (e.g., azacytidine) highlights its potential role as a prognostic biomarker and therapeutic target in myelodysplastic syndromes and acute myeloid leukemia [44].

While HAI-2 protein levels are decreased in prostate cancer (PCa), its mRNA levels do not show a consistent corresponding change, neither is the *SPINT2* promoter hypermethylated. Therefore, its deregulation mechanism in PCa differs from that in other malignancies, probably involving post-translational regulation [45].

Regarding epigenetic regulation in non-cancer contexts, a recent study showed that DNMT1-mediated methylation normally represses *SPINT2* expression in fibroblasts. Loss of DNMT1 results in hypomethylation and upregulation of *SPINT2* gene, which in turn inhibits c-Met signaling and triggers senescence in human fibroblasts [46]. Additionally, increased *SPINT1* promoter methylation has been reported in amnestic mild cognitive impairment and Alzheimer’s disease compared with controls [47]; however, its relationship with dementia severity appears complex and non-monotonic. Histone modifications and chromatin remodeling are other critical epigenetic mechanisms regulating *SPINT2* expression, particularly during immune cell activation. In human macrophages, *SPINT2* functions as a target gene of Interleukin-4 (IL-4) and the transcription factor signal transducer and activator of transcription 6. Upon IL-4 stimulation, the *SPINT2* gene locus undergoes distinct chromatin changes, characterized by the removal of the repressive histone mark histone H3 lysine 27 trimethylation and a significant reduction in nucleosome density [48].

#### 3.4.2. Transcriptional Regulation

Both mouse *Spint1* and *Spint2* are TATA-less/CAAT-less genes. However, no apparent homologous portion was observed between *Spint1* and *Spint2* promoter regions. Only the *Spint1* promoter harbors a complex of Egr-1-3 and Sp1 that are functionally required for robust promoter activity and is proposed to mediate early-response upregulation during tissue injury and regeneration. This implies that *Spint1* and *Spint2* are different in their upstream transcriptional regulation mechanisms [49]. Direct regulation of *Spint1* by the epithelial factor Grainyhead-like-2 (GRHL2) has been demonstrated in a developmental model of mouse submandibular salivary gland (SMG). SiRNA-mediated knockdown of *Grhl2* in ex vivo SMG organ culture reduced *Spint1* mRNA and protein, impaired branching morphogenesis, and disrupted basal lamina organization [50]. Additionally, CDX2 has been reported to serve as a key transcriptional regulator that either positively or negatively regulates the gene expression of *ST14* and *SPINT1* in human intestinal epithelial cells, thereby fine-tuning the expression balance between them, influencing epithelial barrier integrity, and potentially impacting colorectal carcinogenesis [51].

#### 3.4.3. Posttranslational Regulation

Membrane-type 1- matrix metalloproteinase (MT1-MMP) cleaves HAI-1 at the flanking region between the KD-1 and LDLR-like domains, reducing its inhibitory effect. This allows matriptase activation, which in turn converts pro-urokinase-type plasminogen activator (pro-uPA) into active uPA, thereby linking MMP and serine protease systems to promote aggressive carcinoma invasion [52]. Cell-bound MMP-7 cleaves HAI-1 predominantly at Gly^451^ and Leu^452^ and releases the extracellular region of HAI-1 as soluble HAI-1 (sHAI-1). sHAI-1 promotes homotypic aggregation of colon carcinoma cells and facilitates invasive growth. Interestingly, cholesterol sulfate is required for MMP-7–mediated cleavage of HAI-1 to generate soluble HAI-1 (sHAI-1), whereas the subsequent sHAI-1–induced cell aggregation relies on cell-surface proteolytic activity of MMP-7 that is independent of cholesterol sulfate [53].

#### 3.4.4. Regulation by Hypoxia

In epithelial models, hypoxia (1% O_2_ or CoCl_2_) and H_2_O_2_ exposure upregulate *SPINT1* mRNA and cell-surface protein within hours in human lung (HLC-1) and colon (WiDr) carcinoma cells. Promoter mapping localizes the hypoxia/oxidant responsiveness to a short GC-rich segment (−79/−30) carrying an overlapping Egr-1/Sp1 site, and mutation of this element blunts induction. Tumor areas positive for hypoxia-inducible factor-1α (HIF-1α) or lipid peroxidation (measured by 4-hydroxy-2-nonenal levels) tend to co-express HAI-1, linking tissue hypoxia/oxidation to HAI-1 regulation [54]. Macrophage HIF-2α sustains HAI-1 under hypoxia; its deletion reduces *Spint1* expression and HAI-1 secretion and accelerates tumor growth in a breast cancer allograft model. Functionally, hypoxic HIF-2α-deficient macrophage supernatants (lacking HAI-1) permits pro-HGF to stimulate tumor-cell proliferation, whereas hypoxic wild-type supernatants (rich in HAI-1) does not [55]. Similarly, in breast cancer, hypoxia upregulated *SPINT2* mRNA and protein only in HER2 (c-erbB2)–amplified lines (SKBR3, BT474), and HIF-1α knockdown suppressed this effect. Clinically, elevated tumor HAI-2 protein levels correlated with higher T stage, nodal involvement, and increased expression of hypoxia markers (CAIX, HIF-1α). Notably, elevated HAI-2 levels predicts a poorer clinical complete response to neoadjuvant epirubicin, although it does not correlate with overall findings [56].

### 3.5. Interaction with Various Serine Proteases

#### 3.5.1. Matriptase Regulation and Structural Interactions

Studies investigating how HAI-1 physically interacts with its targets may provide insights into the structural requirements for effective inhibition. The tight relationship between matriptase and HAI-1 extends beyond simple inhibition, involving HAI-1′s unconventional function in regulating matriptase trafficking and activation. HAI-1′s LDL receptor class A domain has been shown to be required for matriptase activation, suggesting that HAI-1 may facilitate transactivation before acting as a competitive inhibitor [57]. Matriptase contains both catalytic and non-catalytic domains in its extra-cellular region, the latter referred to as the stem region. When the inhibitory activity of membrane-anchored recombinant HAI-1 (maHAI-1) was tested against two matriptase variants, one containing the full extracellular domain (HL-matriptase) and the other containing only the catalytic domain (L-matriptase), distinct differences were observed. Inhibition of both enzymes by purified maHAI-1 was similar with substrates corresponding to pro-HGF– and latent matriptase–like cleavage sequences, but HL-matriptase was more strongly inhibited than L-matriptase by a substrate mimicking the prostasin cleavage sequence. This result indicates that the stem domain of matriptase appears to enhance HAI-1 inhibition under certain substrate contexts [58]. However, matriptase proteolysis was unexpectedly suppressed in HAI-1-deficient cells, demonstrated by reduced zymogen activation, less shedding of active matriptase, and diminished matriptase-dependent prostasin zymogen activation. This suppression is attributed to both the decreased ability of HAI-1-deficient cells to activate matriptase and the rapid inhibition of nascent active matriptase by HAI-2 or other unknown protease inhibitors [59].

HAI-2 has also been proposed as a physiological regulator of matriptase activity, possibly acting in a redundant or partially redundant manner with HAI-1. HAI-2 displayed potent inhibitory activity toward matriptase, forming sodium dodecyl sulfate-stable complexes with the protease and blocking matriptase-dependent activation of candidate physiological substrates, such as pro-prostasin and cell-surface-bound uPA [60]. This role was supported by the striking co-localization of HAI-2 with matriptase and HAI-1 across epithelial cells in all major organ systems, as revealed by a global, high-resolution mapping of adult tissues. However, HAI-2 expression was also uniquely detected in non-epithelial cells of the brain and lymph nodes, where matriptase is not typically expressed, suggesting its involvement in inhibiting other serine proteases beyond matriptase [60].

#### 3.5.2. Matriptase Zymogen Inhibition and Chaperone Function of HAIs

A further study expanded the focus on matriptase activity to include its zymogen form. Matriptase belongs to a rare subset of serine proteases that displays significant intrinsic activity even in its zymogen form. Both HAI-1 and HAI-2 were found capable of inhibiting the catalytic activity of matriptase zymogen toward peptide substrates, pro-HGF, and zymogen prostasin in a manner similar to their inhibition of activated matriptase. HAI-1 inhibition depends exclusively on KD1, while HAI-2 utilizes both KD1 and KD2. This ability of HAI-1 and HAI-2 to inhibit the intrinsic activity of the matriptase zymogen suggests they function as regulators of matriptase trans-activation and auto-activation [61].

HAI-1 and HAI-2 also retain a chaperone function, promoting matriptase expression and cell-surface translocation. Notably, even very low levels of the inhibitor proteins appear sufficient to support this trafficking function in certain cellular contexts [31]. This chaperone activity is mediated primarily by the intracellular inhibition of undesirable matriptase catalytic activity by KD1. Additionally, the adjacent PKD domain-like internal domain was found essential for HAI-1′s chaperone function, as it is required for the proper folding and membrane trafficking of the inhibitory HAI-1 [62].

#### 3.5.3. Matriptase Activity Control Under Inhibitor Deficiency

In cells lacking sufficient HAI-1, where the HAI-1-to-matriptase ratio is only 1, such as in ovarian cancer or multiple myeloma, matriptase activity may be controlled through alternative mechanisms. When matriptase activation was induced in these low HAI-1 cells, activated matriptase rapidly formed a 140 kDa matriptase homodimer and a 100 kDa complex, in addition to the canonical 120 kDa matriptase-HAI-1 complex. The 140 kDa homodimer is an enzymatically inactive intermediate formed during the autoactivation process, serving as a mechanism to control matriptase activity when the inhibitor is limiting. The 100 kDa complex contains HAI-2-like peptides and other peptides, suggesting it may contain HAI-2 or an unidentified serine protease inhibitor(s) [63].

#### 3.5.4. Prostasin Regulation

Investigating the consequence of HAI-1 deficiency in HaCaT human keratinocytes revealed distinct and opposing impacts on its two main targets, matriptase and prostasin. Matriptase activation was markedly reduced under HAI-1–deficient conditions, as described above (II-3-1). In contrast, HAI-1 deficiency led to an increase in prostasin proteolysis, primarily through enhanced protein expression and zymogen activation. However, this increase in activated prostasin was detected predominantly in complexes with HAI-2, suggesting that the net prostasin enzymatic activity remains under tight control even without HAI-1 [59].

#### 3.5.5. Comparison of HAI-1 and HAI-2 of the Prostasin–Matriptase Cascade

The functional relationship between HAI-1 and HAI-2, particularly in epithelial homeostasis, has been further delineated in using mouse intestinal models. While HAI-1 ablation did not affect the expression or subcellular localization of matriptase in the intestine, loss of HAI-2 resulted in a dramatic reduction in matriptase protein. This loss was linked to accelerated activation and shedding of matriptase caused by uncontrolled prostasin activity. The activation and shedding of matriptase by HAI-2 depletion could be rescued by simultaneous depletion of prostasin levels in Caco-2 cells, confirming the prostasin-dependent nature of this regulation. This finding indicated that HAI-2 plays an essential role in regulating prostasin-dependent matriptase zymogen activation, while HAI-1 primarily regulates the activity of already-activated matriptase [64]. Further context for this proteolytic cascade was found in human milk, where activated matriptase and prostasin were present in complexes with both HAI-1 and HAI-2. In addition to the well-known matriptase-HAI-1 complex, complexes of prostasin-HAI-1 and prostasin-HAI-2 were purified and identified, suggesting that the proteolytic activity of matriptase and prostasin is significant during lactation and tightly controlled by the HAIs. However, in cultured human mammary epithelial cells, HAI-1 appeared to be the predominant inhibitor, with HAI-2 being mainly intracellular [65], suggesting mammary epithelial cells may not be the in vivo source of the prostasin-HAI complexes in milk, or may have lost this capability upon immortalization.

The *SPINT2* mutations primarily cause intestinal defects, manifesting as syndromic congenital sodium diarrhea, reflecting their organ-selective effects despite HAI-2′s broad expression in many tissues. Further research has supported this observation. In Caco-2 cells, HAI-2 deletion results in significantly enhanced and prolonged prostasin proteolytic activity and consumption and depletion of HAI-1 monomer. This depletion of HAI-1 was found to be prostasin-dependent, not matriptase-dependent. While HAI-2 loss increases matriptase and prostasin zymogen activation in both Caco-2 cells and HaCaT keratinocytes, the HAI-1 monomer level remains high in HaCaT cells, suggesting that the intestine’s inherently high prostasin zymogen activation and its strong reliance on HAI-2 for prostasin regulation make Caco-2 cells and the GI epithelium in vivo particularly vulnerable to HAI-2 loss [66].

#### 3.5.6. Subcellular Localization and Inhibitory Function of HAI-1 and HAI-2

The differential control mechanisms exerted by HAI-1 and HAI-2 are largely explained by their distinct subcellular localization patterns. Although both are widely co-expressed, HAI-1 acts as the default inhibitor and is distributed both intracellularly and on the cell surface [67]. In neoplastic B-cells, increasing HAI-1 suppresses extracellular active matriptase proportionally [31]. A further study revealed the HAI-1 likely inhibits active matriptase zymogen in the ER and secretory pathway before it reaches the plasma membrane [61].

HAI-2 acts as a cell-type-selective inhibitor, which is predominately confined to intracellular granules [67]. Increasing HAI-2 fails to achieve the same suppression in neoplastic B-cells, largely because HAI-2 is mainly intracellular [31]. Furthermore, the critical role of subcellular localization demonstrates that HAI-2 acts as a matriptase inhibitor in breast cancer cells but not in immortalized mammary epithelial cells. Although HAI-2 was a more potent matriptase inhibitor than HAI-1 in solution, HAI-2 remained sequestered in intracellular granular/vesicle structures in mammary epithelial cells, thus lacking access to activated matriptase found at cell–cell junctions. In contrast, misrouting of HAI-2 to the cell surface in breast cancer cells allowed it to co-operate with HAI-1 in matriptase inhibition, forming multiple matriptase-HAI-2 complexes [31]. The inhibition of active matriptase zymogen by HAI-2 also occurs in the ER and secretory pathway prior to its arrival at the plasma membrane [61].

#### 3.5.7. Regulation of TMPRSS13 and HAT

The protease activity of TMPRSS13, a member of the TTSP family, was shown to be inhibited by HAI-1. Consistent with the structural findings for matriptase inhibition, a truncated HAI-1 fragment containing KD1 (NK1) demonstrated stronger inhibitory activity against TMPRSS13 than the soluble form containing both Kunitz domains. HAI-2 was also found to inhibit TMPRSS13, with potency comparable to that of NK1 fragment [68].

A further regulatory role of HAI-1 was identified in human airway trypsin-like protease (HAT), a transmembrane serine protease primarily expressed in bronchial epithelial cells. A soluble form of HAI-1 was found to inhibit the protease activity of HAT in vitro. HAT undergoes proteolytic activation in cultured mammalian cells, a process that relies on its own serine protease activity. Notably, co-expression of the full-length transmembrane HAI-1 inhibits the proteolytic activation of HAT, and full-length HAI-1 was observed to associate with the full-length HAT in cells co-expressing them. Functionally, HAT, like other target proteases of HAI-1, can convert pro-HGF to the active form in vitro. These findings suggest that HAI-1 functions as a physiological regulator of HAT by suppressing both its proteolytic activation and its resulting protease activity within the airway epithelium [69].

## 4. Physiological and Developmental Roles

**Figure 2:**
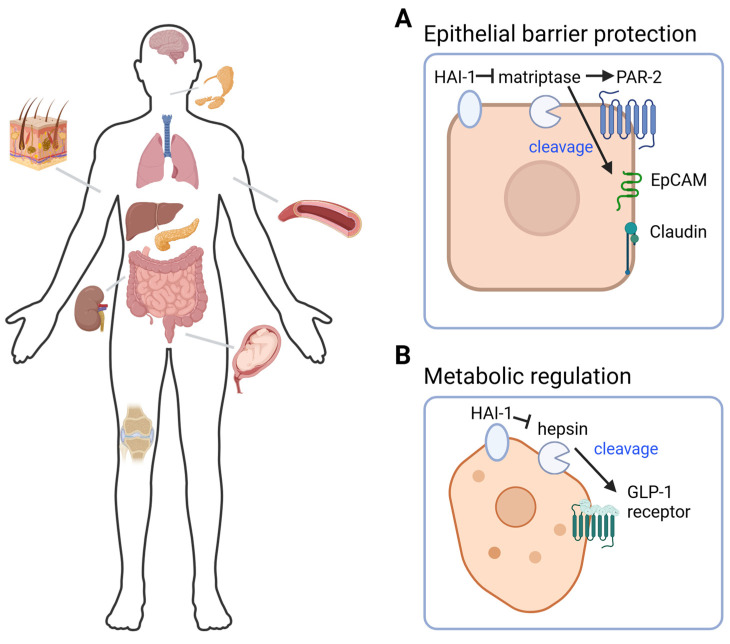
Examples of the developmental and physiological roles of HAI-1 in organ systems. (**A**) Skin: HAI-1 preserves the epidermal barrier by inhibiting matriptase. Loss of HAI-1 causes unchecked proteolysis, which degrades epithelial cell adhesion molecule (EpCAM), downregulates tight junction protein claudin-7, and activates protease-activated receptor-2 (PAR-2)-mediated inflammatory signaling, resulting in ichthyosis. (**B**) Pancreas: HAI-1 regulates glucose metabolism by inhibiting hepsin. Loss of HAI-1 allows hepsin to cleave the GLP-1 receptor on β-cells, impairing insulin synthesis and secretion and causing glucose intolerance. Created in BioRender. Chen, B. (2026) https://biorender.com/qwjmtwi (accessed on 25 January 2026).

Examples of HAI-1′s physiological roles are depicted in Figure 2.

### 4.1. Skin Barrier Formation and Epidermal Differentiation

#### 4.1.1. Molecular Pathogenesis and Physiological Consequences

A study in mice revealed the profound importance of HAI-1 in maintaining epithelial homeostasis. Mice with a global deficiency in *Spint1*, rescued from embryonic lethality caused by placental defects by injecting *Spint1*-deficient embryonic stem cells into wild-type blastocysts to generate chimeras with functional placentas, developed severe postnatal skin abnormalities, including scaly skin reminiscent of ichthyosis, epidermal acanthosis, abnormal hair development, and reduced epidermal barrier function, resulting in a significantly higher rate of fluid loss. The epidermal defects, which included impaired generation of filaggrin monomers and accumulation of filaggrin dimers, suggested deregulation of the matriptase-prostasin proteolytic cascade [10]. Crucially, it was demonstrated that loss of the matriptase-inhibiting function of HAI-1 is the primary cause of these detrimental postnatal effects. HAI-1 and its target protease, matriptase, co-localized in affected keratinized tissues like the epidermis and hair follicle. Furthermore, *Spint1*-deficient mice that possessed low levels of matriptase, due to a hypomorphic mutation in the *St14* gene, survived the neonatal period, were healthy, and displayed normal tissue homeostasis, confirming that matriptase suppression is an essential function of HAI-1 in maintenance of postnatal epidermal tissues [70]. The skin condition resulting from HAI-1 deficiency, which showed enhanced phosphorylation of Akt in keratinocytes, was similar to phenotypes observed when matriptase activity was abnormally reduced, highlighting the critical need for a tight balance in protease activity [10]. When HAI-1 function is insufficient, the integrity of keratinocytes is compromised. Mouse *Spint1*-deleted epidermis showed reduced assembly of keratin intermediate filaments into desmosomes, accompanied by a decrease in desmosome number. This structural deficiency was associated with p38 mitogen-activated protein kinase signaling downstream of protease-activated receptor-2 (PAR-2), which was activated by unregulated matriptase activity resulting from insufficient HAI-1. The addition of a p38 inhibitor or PAR-2 antagonist was shown to restore the normal keratinocyte morphology, suggesting that HAI-1 maintains epidermal integrity by suppressing PAR-2 activation via regulating matriptase activity [71].

The critical balance between protease activity and inhibition observed in murine skin is also essential to human epidermal differentiation, where the matriptase-prostasin cascade is constitutively active and requires tight coupling with HAI-1. Further studies showed more details on the dysregulation of protease cascades and protease-mediated signal transduction caused by deficiency of HAI-1. In human epidermal differentiation, the proteolytic cascade involving matriptase and prostasin is constitutively activated from an early stage. Prostasin activation is dependent on the proteolytic action of matriptase in human keratinocytes. This tight coupling requires that both active matriptase and active prostasin are readily inhibited by HAI-1 binding, ensuring only a very brief window of opportunity for proteolytic activity to act on downstream substrates [72].

Recent findings have identified new substrates for matriptase, epithelial cell adhesion molecule (EpCAM) and trophoblast cell surface antigen 2 (TROP2), both of which are co-expressed in human keratinocytes. Matriptase cleavage of EpCAM and TROP2 triggers the destabilization and lysosomal degradation of these proteins and the associated tight junction proteins claudin-1 and claudin-7 [73]. Similar to the broader protease inhibition context, HAI-1 plays a more important role than HAI-2 in inhibiting the matriptase-mediated cleavage of EpCAM and TROP2 in keratinocytes. The loss of HAI-1 inhibition-induced cleavage and subsequent downregulation of claudin-1 and claudin-7 was eliminated when matriptase was also depleted, proving matriptase’s involvement. Simultaneous knockdown of both HAI-1 and HAI-2 resulted in marked increases in cleavage and claudin loss, suggesting that the inhibitors are partially redundant and cooperate to regulate matriptase in keratinocytes. The effect of HAI-1 deficiency on claudin-1 stability is proposed to be a cause of HAI-1 deficiency-induced ichthyosis [73]. In a genome-wide association study conducted in the Japanese population, the single-nucleotide polymorphism (SNP) rs2278431 was associated with increased corneocyte area, which is an indicator of decreased epidermal turnover, and subsequent analysis suggested this SNP is related to *SPINT2* expression. *SPINT2* knockdown in epidermal keratinocytes decreased proliferative capacity and enlarged corneocyte area in a 3D-reconstructed epidermis model, suggesting that *SPINT2* positively regulates keratinocyte proliferation and epidermal turnover [74].

#### 4.1.2. Context-Dependent Regulation and Spatiotemporal Distribution

The role of these inhibitors is context-dependent and based on the specific location within the skin. In the human pilosebaceous unit, including hair follicles and sebaceous glands, matriptase is highly expressed in proliferative cells, such as basal cells of the epidermis and the matrix cells of hair follicles. Its activation is similarly restricted to these basal and proliferative areas. Conversely, matriptase expression is low or absent in terminally differentiated cells (e.g., inner root sheath, granular layer). The expression of matriptase follows a cycle-dependent pattern, with high levels observed during the anagen and catagen phases of the hair cycle, followed by reduced expression of HAI-1 in the catagen phase [75,76].

In human skin, prostasin exhibits high-level constitutive activation primarily in the granular layer, which is associated with late-stage epidermal differentiation, in contrast to the low-level activation of matriptase in basal cells. This inverse pattern suggests that the functional link between matriptase and prostasin is not as strong in human skin as observed in cultured cell models [77,78]. Furthermore, in human skin, HAI-1 is the major functional inhibitor of matriptase and prostasin. This is because HAI-1 is widely distributed on the cell surface of all three viable epidermal layers, granting it direct access to active proteases. In contrast, HAI-2 is expressed predominantly in the basal and spinous layers but maintains a largely intracellular localization, preventing it from effectively inhibiting cell-surface matriptase or prostasin in the skin [77,78].

This regulatory hierarchy is mirrored in the zebrafish epidermis, where HAI-1a, which is encoded by *Spint1a*, is required for epidermal integrity, but HAI-2 is dispensable for epidermal development. *Spint1a* interacts genetically with EpCAM, a relationship not observed for *Spint2* [79]. Zebrafish mutants carrying an insertion in the *spint1* gene exhibit a phenotype resembling chronic inflammation and human psoriasis, characterized by neutrophil and macrophage infiltration into the fin, coupled with underlying epidermal hyperproliferation [80]. The disruption and hyperproliferation precedes the inflammatory response. This chronic inflammatory phenotype is caused by the reduced expression of *spint1*, which leads to unregulated activity of matriptase 1. Importantly, transient knock-down of matriptase 1 rescued the *spint1* mutant phenotypes, confirming matriptase 1 as a key effector downstream of *spint1* in regulating epidermal proliferation and inflammation [80]. Live imaging of this chronic inflammatory state revealed that neutrophils displayed a biased random walk migration with frequent pauses, in sharp contrast to the directed chemotaxis observed during acute injury [80]. The mechanism underlying this neutrophil abnormality remains unclear, but is thought to result from dysregulation of the complex interplay of extracellular matrix and inflammatory mediators caused by *spint1* deficiency.

### 4.2. Gastrointestinal Tract: Intestinal Barrier and Mucosal Repair

HAI-1 and HAI-2 are expressed abundantly throughout the human and mouse gastrointestinal (GI) tract [81,82]. Early research, particularly focusing on mucosal regeneration, established that HGF/scatter factor (SF) plays an important role in repairing injured GI mucosa by promoting the proliferation and migration of epithelial cells, a process dependent on HGFA [81,82]. Furthermore, a study focusing on intestine-specific deletion of *Spint1* in mice demonstrated that HAI-1 is essential for maintaining intestinal epithelial integrity. Loss of intestinal HAI-1 led to histologic abnormalities, particularly in the proximal aspect of the colon, including increased epithelial cell apoptosis and turnover, as well as increased intestinal permeability, as evidenced by elevated plasma levels of orally administered fluorescein isothiocyanate dextran-dextran. Mechanistically, *Spint1* deficiency resulted in a dysregulated subcellular localization pattern of its cognate protease, matriptase, shifting its distribution towards a diffuse cytoplasmic staining pattern in disorganized crypt epithelial cells. Consequently, mice lacking intestinal HAI-1 exhibited enhanced susceptibility to dextran sodium sulfate-induced experimental colitis, a model of inflammatory bowel disease, suggesting a crucial role for HAI-1 in maintaining colon epithelial integrity and susceptibility to injury [11].

A study using cell-based models hypothesized that the loss of HAI-2 leads to unrestrained activity of the protease matriptase, which subsequently cleaves EpCAM after Arg80. Since EpCAM stabilizes the tight junction protein claudin-7, its cleavage causes both EpCAM and claudin-7 to be targeted for internalization and lysosomal degradation, thereby compromising the epithelial barrier. This proposed pathway was supported by findings that congenital tufting enteropathy-associated HAI-2 mutant proteins, such as Y163C or G168S, showed a reduced ability to inhibit matriptase and stabilize claudin-7 [83]. However, a conflicting report emerged regarding the primary target of HAI-2 in human enterocytes. Biochemical analysis demonstrated that HAI-2 selectively inhibits prostasin in Caco-2 cells and human intestinal tissue, forming complexes predominantly with activated prostasin, not matriptase. Prostasin and HAI-2 immunoreactivity localizes intensely near the brush borders of villus epithelial cells, whereas matriptase and HAI-1 localize mainly to the lateral plasma membrane. Therefore, the loss of HAI-2 is expected to result in aberrant regulation of prostasin, leading to sodium loss [84].

Additionally, in intestinal cell lines, CDX2 has been reported to negatively regulate *SPINT1* and either positively or negatively regulate *ST14* (encoding matriptase) in a context-dependent manner through binding to their enhancer regions [51].

### 4.3. Reproduction and Development: Placental Function and Embryogenesis

#### 4.3.1. The Critical Role of HAI-1 in Placental Labyrinth Development and Basement Membrane Integrity

The physiological function of HAI- is critical in reproduction and embryonic development, particularly in the formation and maintenance of the placenta. An early clinical investigation in women revealed that *SPINT1* gene expression showed a gradual increase in villous tissue from 6 to 9 weeks of pregnancy, supporting its importance in early embryo development [85]. In human placenta, HAI-1 was localized specifically to Langhans cells of villous cytotrophoblasts in the chorionic villi tissue, which are considered proliferating trophoblastic stem cells, while syncytiotrophoblasts were generally negative, and extravillous trophoblasts showed markedly decreased HAI-1 immunoreactivity. The presence of HAI-1 in this specific location suggested a potential role in trophoblast proliferation and placenta development [86].

The essential developmental role of HAI-1 was firmly established through mouse knockout studies. Homozygous *Spint1*-deficient mice experienced embryonic lethality around embryonic day (E) 10.5 to E11.5 due to failed placental development and function [12]. This lethality was attributed primarily to the severely impaired formation of the labyrinth layer, a key site for nutrient transport, while other placental layers, including spongiotrophoblast and giant cell layers, were formed normally. The defects in the labyrinth include a complete failure of vascularization and attenuated trophoblast branching morphogenesis [12,87].

Further mechanistic studies showed that the root cause of the placental failure in *Spint-1*-deficient mice was the disruption of basement membranes within the placental labyrinth. Immunofluorescent staining revealed that in *Spint1* deficient placentas, the basement membrane components collagen IV and laminin, which normally exhibits a regular linear distribution separating the chorionic trophoblasts from the allantoic mesoderm, were severely disrupted. Their deposition appeared patchy and discontinuous, with laminin immunoreactivity specifically displaying an irregular, intense punctate pattern rather than a continuous sheet [87,88,89]. This finding correlated directly with the defects in vascularization [87]. The expression of *Spint-1* co-localized with its protease targets, matriptase and prostasin, in the labyrinthine trophoblast cells near the basement membranes [87]. In fact, the placental labyrinth was found to be the only location where matriptase, prostasin, and HAI-1 are all co-expressed in wild-type embryos and yolk sacs [87]. The absence of HAI-1 resulted in hypothesized uncontrolled proteolytic activities of matriptase and prostasin, which likely caused the degradation and disruption of the basement membrane components [87]. A subsequent genetic ablation study confirmed that matriptase inhibition by HAI-1 is the crucial, non-redundant function for placental development. Loss of HAI-1 resulted in the disruption of the epithelial integrity of matriptase-expressing chorionic trophoblasts, which was characterized by disorganized laminin deposition and altered expression of epithelial markers like E-cadherin and β-catenin. Critically, the placental and embryonic defects in *Spint1*-deficient mice were completely rescued by the simultaneous genetic ablation of matriptase [90].

When *Spint1* knockdown (KD) was performed in BeWo cells, which is a human choriocarcinoma trophoblast cell line, the amount of cellular laminin protein increased, while the activity associated with laminin degradation in the culture supernatant decreased. A key finding regarding protease localization was that cell-associated matriptase was significantly decreased in the KD cells, despite the mRNA level for matriptase remaining unaltered. This outcome suggests that HAI-1 is critically required for the cell surface localization of matriptase in trophoblasts, and its absence leads to an enhanced release or dislocation of matriptase [89]. Taken together, the BeWo knockdown and the in vivo placental data may represent different readouts of the same underlying defect. Loss of HAI-1 destabilizes and mislocalizes matriptase at the trophoblast surface, impairing the polarized basolateral secretion and pericellular processing of laminin. As a result, laminin accumulates within cells and is inefficiently incorporated into the basement membrane, leading in vivo to the irregular, punctate, and discontinuous laminin staining pattern characteristic of *Spint1*-deficient placentas.

The transcription factor Grhl2 was identified as upstream regulator of *Spint1* expression in placental cells. In *Grhl2*-deficient mouse embryos, defects in basal chorionic trophoblast (BCT) cell polarity and basement membrane integrity resulted in severe disruption of labyrinth branching morphogenesis, leading to embryonic lethality similar to that observed in *Spint1* knockout mice. Grhl2 directly regulated a network of genes, including *Spint1*, suggesting that the Grhl2-dependent *Spint1* expression, and the ensuing integrity of the BCT layer mediated by HAI-1, is central to proper labyrinth morphogenesis [88]. In addition, a study in pigs suggested that HAI-1 regulates placental folds development by controlling trophoblast cell proliferation and invasion. *Spint1* expression was strong in high columnar trophoblast cells which have low proliferative and invasive capacities, but low in cuboidal trophoblast cells which have high proliferative and invasive capacities [91].

#### 4.3.2. HAI-2 Specific Functions and the Complex Interplay of the Protease Network

Similar to *Spint1*, *Spint2* deficiency in mice also resulted in embryonic lethality, but with additional severe developmental defects including defects in neural tube closure. *Spint2* deficiency also caused placental labyrinth abnormalities associated with a pronounced defect in the polarization of the chorionic epithelium and delocalized laminin deposition [92]. The embryonic demise and placental defects in *Spint2*-deficient mice were primarily caused by unregulated matriptase activity, as the simultaneous inactivation of matriptase restored placental development and embryonic survival. However, the elimination of matriptase only partially reduced the frequency of neural tube defects in *Spint2*-deficient mice, indicating that HAI-2 likely regulates additional proteases essential for neural development [92]. Further complexity was revealed in the interplay between these two inhibitors and their targets. A combined analysis showed that matriptase, HAI-1, and HAI-2 form a complex network, and the balance of their inhibitory activity is delicate. Specifically, combined heterozygosity for *Spint1* and *Spint2* was incompatible with term development, but triple heterozygosity, including *St14* (matriptase) heterozygosity, restored normal development. This suggested a partial redundancy between HAI-1 and HAI-2 in regulating matriptase [92].

The mechanism by which matriptase and prostasin interact was further clarified by testing the role of prostasin proteolytic activity. Prostasin catalytic activity was found to be required for matriptase zymogen activation during placental differentiation, evidenced by the absence of the active two-chain matriptase in placentas expressing catalytically inactive prostasin. However, a striking contrast was observed regarding overall survival: while prostasin null (KO) mice exhibited partial embryonic and complete perinatal lethality, mice expressing catalytically inactive prostasin (S238A) were fully viable with normal prenatal and postnatal survival. This unexpected finding demonstrated that the overall prostasin-dependent functions in embryonic survival were independent of its proteolytic activity [93]. Moreover, the loss of prostasin proteolytic activity was not sufficient to rescue the embryonic lethality in *Spint1* or *Spint2*-deficient mice. This suggested that the embryonic lethality associated with *Spint1* or *Spint2*-deficient is mediated by non-proteolytic functions of prostasin [93]. Crucially, the absence of HAI-1 completely compensated for the loss of all prostasin functions, including both proteolytic and non-proteolytic, allowing *Spint1*/prostasin double-deficient mice to achieve normal prenatal and postnatal survival, reinforcing the concept that HAI-1 primarily acts by controlling matriptase [93].

Regarding the expression of HAI-2 in reproductive tissues, HAI-2 was found strongly expressed in the human testis, predominantly localized in primary spermatocytes [94]. Interestingly, the size of *SPINT2* mRNA in the testis (1.2 kb) was found to be shorter than in other tissues, such as the placenta (1.5 kb), suggesting distinct transcriptional or splicing regulation for *SPINT2* during spermatogenesis [94].

### 4.4. Liver: HGFA Regulation and Iron Homeostasis

HAI-1 is a regulator of HGFA, which is produced by hepatocytes in an inactive form and is activated primarily by thrombin or factor Xa in response to tissue injury [95]. Furthermore, it has been demonstrated that hepsin, a membrane-associated serine protease, also efficiently activates pro-HGF at the expected Arg494–Val495 peptide bond. This activity was comparable to that of HGFA [96]. Critically, HAI-1B, which is a splice variant of HAI-1, and HAI-2 were identified as potent inhibitors of hepsin activity. Specifically, the inhibition mediated by HAI-1B is attributed entirely to KD1, whereas KD2 was non-functional in this context [96].

Concerning iron homeostasis, Matriptase-2 (MT2), which is expressed predominantly in the liver, plays a key role in suppressing the iron-regulatory hormone, hepcidin. HAI-2 was identified as a cell surface inhibitor with high inhibitory potential against MT2 [97]. HAI-2 modulates hepcidin expression by abrogating the MT2-mediated suppression of the hepcidin-encoding gene. This mechanism was initially suggested to involve HAI-2 suppression of MT2-mediated cleavage of membrane-bound hemojuvelin, a BMP co-receptor, thereby inhibiting BMP–SMAD signaling, which is the central pathway driving hepcidin transcription. [97]. However, later research indicated a more complex regulation of MT2-hepcidin axis and a more nuanced role for HAI-2 within it. While *Spint2* was expressed in mouse hepatocytes alongside *Mt2*, hepatocyte-specific ablation of HAI-2 in mice resulted in only a marginal impact on systemic iron homeostasis and failed to significantly affect the iron-mediated regulation of hepcidin expression. This finding suggested that MT2 regulates basal hepcidin levels primarily through a nonproteolytic mechanism, implying that the proteolytic inhibition function of HAI-2 in this context is limited [98]. Alternatively, it may reflect the presence of compensatory mechanisms in the hepcidin regulatory network.

### 4.5. Renal Function

In normal kidney tissue, both HAI-1 and HAI-2 mRNA are abundantly expressed, predominantly by the epithelial cells of the uriniferous tubules, followed by the collecting duct and glomerulus. Specifically, *SPINT1* mRNA levels appeared higher in proximal tubules compared to distal tubules, while *SPINT2* expression was comparable in both proximal and distal tubules [99]. Conversely, HGFA mRNA was scarcely detectable in the normal kidney [99]. In addition, the essential roles of these inhibitors in development are underscored by the fact that targeted disruption of the *Spint2* gene in mice resulted in embryonic lethality [99].

More recently, research focused on chronic kidney disease identified matriptase as being induced predominantly in podocytes in mouse models of adriamycin nephropathy. This induction was associated with an imbalance favoring matriptase over its cognate inhibitor, HAI-1. Mechanistically, matriptase mediates pathogenic damage by proteolytically cleaving podocin at Arg50. Consistent with its protective role, the conditional depletion of HAI-1 in podocytes exacerbated the injury in the mouse model. Thus, HAI-1 is considered essential for protecting podocytes by blocking this matriptase-mediated cleavage [100].

### 4.6. Epithelial Function in Lungs

In normal human pulmonary tissue, *SPINT1* is expressed in the bronchial respiratory epithelium, predominantly in basal cells, and weakly in ciliated columnar epithelial cells and alveolar epithelial cells [14]. However, in response to tissue injury and severe inflammation, HAI-1 expression is significantly upregulated. Interestingly, in ciliated epithelial cells of the bronchioles near areas of severe damage (e.g., adjacent to invading carcinoma or inflammation), HAI-1 exhibited a distinct apical translocation from its usual basolateral surface localization. This apical localization might result from transcytosis, suggesting that HAI-1 interacted with ciliated epithelium-specific cognate proteinases, such as prostasin, HAT, or TMPRSS11D, which are localized to the apical surface and regulate epithelial sodium channel (ENaC) activity [14].

HAI-1 and HAI-2 are key regulatory factors for epithelial sodium channel (ENaC) activity. ENaC is stimulated constitutively by trypsin-family serine peptidases, known as channel-activating peptidases (CAPs), including prostasin, TMPRSS4, and matriptase, a process revealed by the inhibitory effect of broad-spectrum inhibitors of peptidases, such as aprotinin and HAI-2 [101]. Prostasin is considered the major basal regulator of ENaC activity in airway cells. HAI-2 was a potential endogenous modulator that inhibits CAP activity and ENaC-mediated sodium hyperabsorption in cultured airway cells [101]. Additionally, HAI-1B, can also inhibit prostasin. Targeting prostasin-like peptidases with HAIs is suggested to hold therapeutic potential for reversing sodium hyperabsorption relevant to lung pathology in cystic fibrosis [101].

### 4.7. Cartilage and Joints

While a preliminary microarray screen identified *SPINT1* as a gene downregulated by SRY-box transcription factor 9 (SOX9) in a human chondrocytic cell line, this regulation was not confirmed upon validation. Subsequent analysis in primary human articular chondrocytes subjected to dedifferentiation in monolayer culture, where endogenous SOX9 levels progressively decreased, the expression of *SPINT1* also decreased, showing a correlation with SOX9 levels [102]. Critically, analysis of osteoarthritic (OA) cartilage, a condition characterized by reduced SOX9 expression compared to healthy tissue, demonstrated that the expression of *SPINT1* was significantly lower in OA cartilage, supporting the functional link between SOX9 and *SPINT1* levels in joint health [102].

### 4.8. Pancreatic Islets: Glucose Homeostasis

The most recent finding highlighted a specific developmental and physiological role for *Spint1* in pancreatic function. *Spint1* expression was observed in the embryonic pancreatic epithelium as early as E12.5 in mice [103]. This study revealed that pancreas-specific *Spint1* deficiency in mice led to glucose intolerance, diminished pancreatic islet size and mass, and impaired insulin production [103]. *Spint1* functions as a crucial cognate inhibitor of hepsin in pancreatic beta cells. Mechanistically, the depletion *Spint1* results in hepsin overactivity, which causes the proteolytic cleavage of the Glucagon-like peptide 1 receptor (GLP1R) [103]. This cleavage suppresses GLP1R signaling, thereby reducing the production of cyclic AMP and consequently downregulating the transcription factor MAFA and insulin expression. Thus, *Spint1* is proposed to maintain MAFA-dependent insulin production by preventing hepsin-mediated GLP1R cleavage, suggesting that SPINT1 and hepsin may represent promising therapeutic targets for diabetes [103].

### 4.9. Nervous System and Brain Development

*Spint1* and *Spint2* are expressed in neural progenitor cells (NPCs), particularly in nestin-positive cells derived from the striatal anlage of the developing rat brain [104]. Mechanistically, HAI-1 and HAI-2 were found to regulate NPC proliferation and fate; overexpression of either HAI-1 or HAI-2 decreased cell proliferation in cultured NPCs. Correspondingly, silencing or antibody blockade of HAI-1 or HAI-2 increased proliferation [104]. HAI-1 also specifically influenced differentiation, increasing the number of glial fibrillary acidic protein-expressing cells, such as astrocytes in culture. *Spint1* overexpression in vivo reduced cell proliferation in the neuroepithelium of E15-old mice and promoted astroglia formation in neonatal mice [104]. The regulation of *Spint1* and *Spint2* mRNA levels in NPCs was linked to BMP signaling, as bone morphogenetic protein-2 (BMP-2) and BMP-4 increased their expression. The BMP-mediated decrease in NPC cell division was partially blocked by the downregulation of *Spint1* and *Spint2* mRNA [104]. Furthermore, overexpression of human *SPINT1* was found to regulate rat glial cell differentiation, mediating some of the BMP effects on cell fate. Interestingly, canonical HGF signaling appeared irrelevant in this context, as the HGF receptor c-Met was hardly detectable, and exogenous HGF did not influence proliferation [104].

Most recently, the expression of *Spint1/SPINT1* was confirmed in the epithelial cells of the choroid plexus (CP) in mouse and human brains. The corresponding mRNA was also detected in mouse CP tissues derived from the lateral and fourth ventricles. In human CP epithelial cells (CPE), HAI-1 immunoreactivity was noted primarily in the cytoplasm and typically localized in cells expressing E-cadherin and Smad-interacting protein 1 (SIP1), a transcriptional repressor for the E-cadherin-encoding gene [105]. The study suggested that HAI-1 may be vital for the survival of enlarged CPEs, which may be damaged by aging and neurodegenerative diseases [105].

An early study demonstrated the presence of HAI-1 in the central nervous system, localizing it to white matter astrocytes in neurologically normal persons and patients with Alzheimer’s disease (AD) and cerebral infarction [106]. Astrocytes are known to produce HGF and HGFA, and HAI-1 appears to regulate the HGF pathway in the pericellular microenvironment of these cells [106]. While HAI-1 immunoreactivity in AD tissues was weaker compared to controls, suggesting increased consumption or turnover in AD, the expression intensity of HAI-1 mRNA remained similar across groups [106]. Conversely, intense HAI-1 immunolabeling was observed in infarct areas, potentially reflecting HGF’s neurotrophic function in response to cerebral injury, together with a compensatory feedback increase in HAI-1 aimed at restraining excessive pericellular protease activity during acute injury [106].

### 4.10. Vascular System: Angiogenesis and Protection

HAI-1 has been analyzed in human endothelial cells, revealing differential localization across the vascular tree. HAI-1 was observed in the endothelial cells of capillaries, venules, and lymph vessels, but it was poorly stained or hardly detectable in arterial endothelial cells (e.g., those derived from the aorta) [107]. These HAI-1-positive endothelial cells also expressed the HGF/SF receptor, MET, suggesting HAI-1 plays a regulatory role in the HGF/SF-MET signaling axis relevant to angiogenesis. Endothelial HAI-1 may function as both an inhibitor of HGFA and, paradoxically, as a reservoir for activated HGFA on the cell surface, which may be crucial for the subsequent angiogenesis phase initiated by tissue injury and inflammation [107].

The role of HAI-2 has been investigated in the pathogenesis of thoracic aortic dissection (TAD). Bioinformatics analysis revealed that *SPINT2* expression is prominently decreased in ascending aorta tissues from TAD patients, where it is mainly localized in aortic smooth muscle cells (SMCs) [108]. This downregulation of *SPINT2* was recapitulated in vitro by treating SMCs with platelet-derived growth factor BB (PDGF-BB). *SPINT2* acts protectively against TAD progression by suppressing proliferation, migration, and phenotypic switching of SMCs [108]. Overexpression of *SPINT2* reduced the proliferation in Ki-67-positive SMCs and inhibited their migration in wound healing assay [108]. Furthermore, *SPINT2* inhibited the PDGF-BB-induced increase in active MMPs, specifically MMP-2 and MMP-9. Critically, *SPINT2* overexpression mitigated the PDGF-BB-induced phenotypic switching of SMCs from a contractile state to a synthetic type [108]. This beneficial effect of *SPINT2* on SMC phenotype and migration was mediated through inhibition of the extracellular signal-regulated kinase signaling pathway [108].

### 4.11. Salivary Gland: Branching Morphogenesis 

HAI-1 has also been investigated in the context of salivary gland development. In the developing mouse submandibular salivary gland (SMG), Grhl2 regulates *Spint1* expression. Knockdown of *Grhl2* in cultured SMG severely retarded epithelial growth and branching morphogenesis, a defect concomitant with suppressed *Spint1* mRNA and protein expression. Chromatin immunoprecipitation followed by qPCR confirmed that Grhl2 protein directly binds to the *Spint1* gene [50]. Crucially, the addition of recombinant HAI-1 to the culture medium largely overcame the suppressive effects of *Grhl2* knockdown on SMG epithelial development and restored the continuous deposition of laminin, a key component of the basal lamina. This suggested that Grhl2-regulated *Spint1* expression is critical for organizing the basal lamina and controlling branching morphogenesis in the developing SMG [50].

## 5. Roles and Mechanisms in Cancer Progression

**Figure 3:**
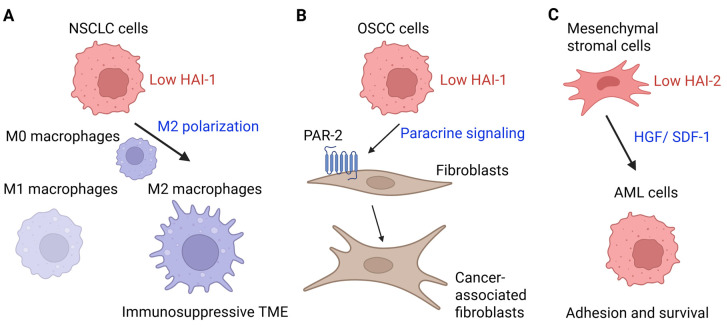
HAI-1 and HAI-2 orchestrate TME crosstalk and immune modulation. (**A**) NSCLC: The loss of HAI-1 leads to an immunosuppressive TME characterized by M2-dominant macrophage infiltration and impaired M1 polarization. (**B**) OSCC: Downregulation of HAI-1 at invasion fronts triggers the paracrine activation of PAR-2 on CAFs, enhancing their migration and tumor-promoting activity. (**C**) AML: HAI-2 downregulation in MSCs increases the secretion of HGF and SDF-1, thereby promoting malignant cell adhesion and survival. Created in BioRender. Chen, B. (2026) https://BioRender.com/rrf2jc9 (accessed on 25 January 2026).

**Figure 4:**
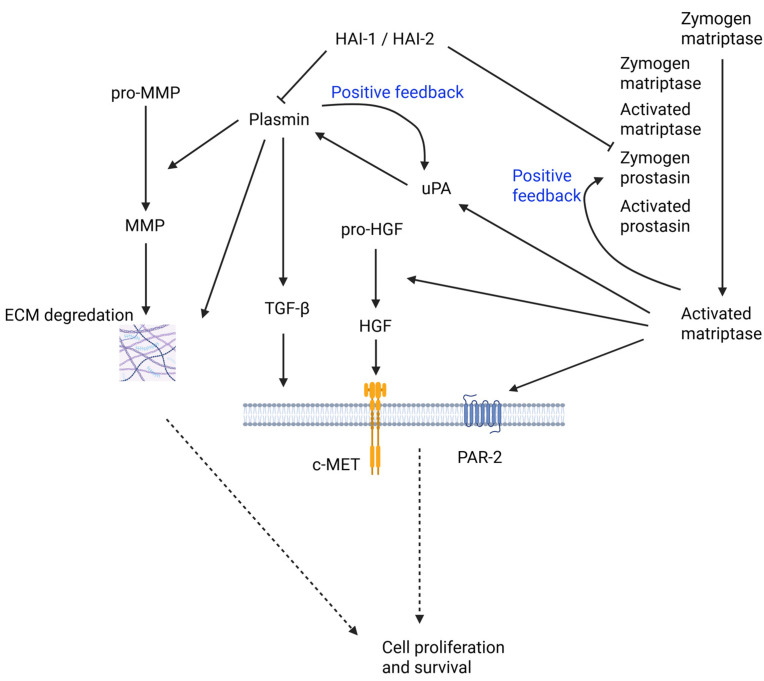
The oncogenic signaling pathways regulated by HAI-1 and HAI-2. Deficiency of HAI-1 and HAI-2 releases inhibitory constraints on the activities of membrane-anchored serine proteases, leading to the activation of matriptase, prostasin, and plasmin. Activated matriptase, which undergoes autoactivation and can also be activated by prostasin, converts pro-HGF into its active form, thereby stimulating c-MET–mediated cell growth and survival. Concurrently, matriptase cleaves PAR-2, triggering downstream signaling pathways that further promote cell growth and survival. In addition, matriptase activates the uPA system, which activates plasmin and is further amplified through a plasmin-dependent positive feedback loop. Plasmin not only converts a pro-MMP to an MMP, leading to ECM degradation, but also directly degrades ECM components. Moreover, plasmin activates latent TGF-β. Both ECM degradation and active TGF-β ultimately converge to promote cell proliferation and survival. Solid arrows indicate direct activation or enhancement; dashed arrows indicate indirect enhancement involving multiple steps. Created in BioRender. Chen, B. (2026) https://BioRender.com/m2mg30s (accessed on 27 January 2026).

HAI-1 and HAI-2 are critically involved in carcinogenesis, especially in cancer progression. Their roles in various cancers and the related molecular mechanisms are summarized in Table 1 and Table 2. Shown in Figure 3 are several crucial and representative examples of their roles in the tumor microenvironment (TME). Figure 4 illustrates the roles of HAI-1 and HAI-2 in regulating oncogenic signaling pathways.

The key facts of their involvement in cancer are highlighted below. Both HAI-1 and HAI-2 can suppress the activity of TTSPs such as matriptase, prostasin, and hepsin, which are known to play tumor-promoting roles in multiple cancers, making their dysregulation implicated in tumor initiation and progression. HAI-1 is generally downregulated in various cancers, including breast [32,109,110,111,112,113,114], prostate [45,115,116,117,118,119,120], gastric [41,121,122], and pancreatic [123,124] cancers. Its loss correlates with enhanced epithelial–mesenchymal transition (EMT), increased invasive potential, and poor prognosis [123,124]. The silencing of HAI-1 often occurs through promoter hypermethylation, leading to unregulated activation of downstream pathways such as HGF/c-MET [122] and PAR-2/NF-κB [125,126]. These pathways contribute to tumor growth, metastasis, and inflammatory responses. Notably, loss of HAI-1 has also been associated with immune evasion, including M2 macrophage polarization, in NSCLC [127].

In contrast, HAI-2 exhibits a more context-dependent role. While it also generally functions as a tumor suppressor by regulating TTSPs and preserving epithelial integrity, in some cancers, such as breast cancer [32,56,96,110,111,112,114] and oral squamous cell carcinoma [128], HAI-2 is paradoxically upregulated and may contribute to tumor progression. This dual potential may stem from tissue-specific upstream regulation, unique subcellular localization, and distinct target protease signatures.

Collectively, dysregulation of HAIs, frequently due to epigenetic process, promoting oncogenesis mainly through protease-dependent mechanisms. Therapeutically, their restoration, either through epigenetic reactivation or recombinant inhibitor mimetics, could offer promising strategies in cancer treatment. Additionally, the ratio of protease to HAI levels has been proposed as a dynamic biomarker in precision oncology [37,120,121].

**Table 1:** The roles of HAI-1 in cancers.

Cancer Type	Expression Pattern	Key Molecular Mechanism	Clinical and Prognostic Outcomes	References
Oral squamous cell carcinoma (OSCC)	downregulated/reduced at invasion fronts	Induction of EMT following loss of expression Promotion of cancer-associated fibroblast (CAF) migration via paracrine PAR-2 activation	Reduction significantly correlating with an increased risk of lymph node metastasis; PAR-2 positive CAFs predicting a shorter disease-free survival.	[89,125,129]
Tongue squamous cell carcinoma	decreased at infiltrative fronts	Orchestration of growth factor activation involved in lymph angiogenesis and invasion by deficiency Enhanced activation of fibroblast-derived pro-HGF Increase in plasminogen-dependent plasmin generation	Decrease significantly correlating with the presence of lymphatic invasion.	[130]
Prostate carcinoma (PCa)	downregulated (progressive Loss)	Development of a more aggressive tumor phenotype following loss Mediation through matriptase-regulated uPA signaling Involvement of PAR-2 signaling pathways.	Progressive loss observed with increasing tumor grade; low HAI-1 expression as a significant predictor for poor prognosis; HAI-1–to–matriptase ratio serving as a biomarker for PCa progression.	[45,115,116,117,118,119,120]
Gastric cancer	downregulated (significantly lower overall); paradoxically enhanced at the invasion front	MACC1 promoting pro-HGF proteolysis and c-MET phosphorylation by transcriptionally inhibition Negative correlation with invasion and lymph node metastasis by inhibiting HGF	Decreased HAI-1 and a lower HAI-1:SNC19/matriptase ratio correlated with more advanced stages and lymph node-positive gastric cancer.	[41,121,122]
Colorectal cancer	downregulated (decreases significantly); paradoxically enhanced at the invasion front	Enhancement of NF-κB activation via PAR-2 signaling following loss Association of downregulation with DNA hypermethylation Mediation of cetuximab resistance through an autocrine HAI-1/protease/HGF/MET axis.	Inverse correlation with the progression of the adenoma-carcinoma sequence; hypermethylation of *SPINT1* correlating with poor prognosis and a decrease in disease-free survival.	[126,131,132,133,134,135]
Non-small-cell lung carcinoma (NSCLC)	loss/downregulated.	Induction of an immunosuppressive tumor microenvironment upon loss of expression Skewing of macrophage polarization toward an M2-dominant phenotype, with impairment of M1 macrophage polarization Inverse association with HGF mRNA expression Sensitization to c-MET inhibitor–mediated growth inhibition upon γ-catenin–induced expression	Loss serving as an independent predictor of poor prognosis, exhibiting a worse median survival; loss frequently detected in patient specimens (60%).	[127,136]
Renal Cell Carcinoma (RCC)	downregulated	Functioning as a marker of epithelial differentiation Correlation of downregulation with tumor de-differentiation Concurrent decrease in expression with enhanced HGFA levels and proteinase activities	Consistently and significantly decreased mRNA levels in RCC tissues; particularly noticeable downregulation in RCC presenting sarcomatoid change.	[99,127],
Ovarian cancer	downregulated	Inhibitory regulation of matriptase expression and hepsin expression Induction of apoptosis	Lower level as a significant predictor for poor prognosis concerning both disease-free survival and overall survival.	[96,137]
Uterine leiomyosarcoma (LMS)	downregulated	Anti-tumor effects mediated by reduced HGFA expression Downregulation of matriptase and hepsin expression Induction of apoptosis and necrosis	Low levels significantly predicting poor prognosis.	[138]
Endometrial cancer	downregulated	Inhibition of cell proliferation upon overexpression Reversal of EMT Upregulation of E-cadherin and Slug expression Downregulation of vimentin, SIP1, Snail, and Twist expression Modulation of estrogen receptor and progesterone receptor signaling	Negative association of levels with lymph node metastasis and lymphovascular space involvement; low HAI-1 predicting a poor prognosis in terms of both disease-free and overall survival.	[139]
Non-Hodgkin B-cell lymphoma	absent/low	Facilitation of free active matriptase generation in lymphoma cells upon deficiency Promotion of active matriptase shedding Activation of pro-HGF and the uPA system	Exogenous expression of *SPINT1* significantly suppressing the proliferation of neoplastic B Cells.	[140]
Hepatocellular carcinoma (HCC)	upregulated	Elevation of expression driven by promoter hypomethylation Correlation of hypomethylation status with poor differentiation	Upregulation associated with poor outcomes, including multiplicity, microscopic vascular invasion, and advanced tumor stage; its positivity functioning as an independent prognostic factor for reduced overall survival and disease-free survival rates.	[33,141]
Breast cancer	upregulated	Association of high expression levels with promoter hypomethylation Downmodulation by hepsin overexpression Augmentation of HGF/MET signaling Requirement of high expression for survival in specific cell lines	High levels associated with poor long-term patient outcome, such as decreased OS, RFS, and DMFS; co-expression of HAI-1 and matriptase predicting a worse outcome.	[32,109,110,111,112,113,114]
Thyroid cancer (TC)	upregulated	Elevation of expression in BRAFV600E-like papillary thyroid carcinoma compared with RAS-like tumors	Enhanced expression positively correlating with aggressive features, including extrathyroidal invasion, lymphovascular invasion, lymph node metastasis, advanced TNM stage, and a higher risk of recurrence.	[142]
Bladder cancer	downregulated; elevated *SPINT1* expression in HIF-2-altered tumors	Essential regulation of MET phosphorylation Contribution of elevated expression in HIF-2–altered tumors to a highly immunosuppressive microenvironment	Low *SPINT1* expression combined with high matriptase expression displaying a significantly poorer prognosis; elevated expression linked to an increased expression in genes promoting immune evasion.	[143]
Pancreatic carcinoma	downregulated or loss	Promotion of EMT following knockdown Induction of invasiveness via activation of the matriptase/PAR-2 axis Localization of anti-metastatic effects to the functional KD1 domain	Its loss promoting metastatic pulmonary colonization in an experimental nude mouse assay.	[123,124]
Glioblastoma (GBM)	complex: pro-malignant or tumor suppressive dependent on the membrane structure	Dependence of pro-malignant activity on membrane structure Modest reduction in invasiveness upon overexpression in U251 cells	Overexpression of *SPINT1* resulting in significantly enhanced tumorigenicity in vivo.	[133]
Skin cutaneous melanoma (SKCM)	deficiency/genetic alterations/high expression	Facilitation of oncogenic transformation upon deficiency Regulation of tumor immune microenvironment crosstalk Correlation of high expression with tumor-associated macrophage infiltration	Genetic alterations in *SPINT1* correlating with poor patient prognosis; deficiency accelerating the onset of SKCM.	[136,144]

OS: overall survival, RFS: recurrence-free survival, DMFS: distant metastasis-free survival.

**Table 2:** The roles of HAI-2 in cancers.

Cancer Type	Expression Pattern	Key Molecular Mechanism	Clinical and Prognostic Outcomes	References
Prostate carcinoma (PCa)	downregulated/progressive loss	Primary regulator of matriptase activity Inhibition of TMPRSS2-mediated pro-HGF activation Suppression of ECM degradation and cell invasion Association of downregulation with *SPINT2* promoter hypermethylation Association of downregulation with post-translational degradation	Its loss pronounced in the most poorly differentiated tumors (Gleason score 8–10). TMPRSS2/HAI-2 ratio increasing in PCa with advanced stages; circulating HAI-2 levels inversely associated with PCa risk.	[27,45,96,118,119,120,145]
Gastric cancer	downregulated/undetectable	Epigenetic inactivation via promoter hypermethylation Functioning as a tumor suppressor gene Inhibition of cell proliferation and induction of apoptosis upon ectopic expression	Hypermethylation correlating with poor differentiation and metastasis in primary tumors.	[41]
Glioblastoma (GBM)/High-grade glioma (HGG)	undetectable/reduced	Association of downregulation with promotor hypermethylation Inhibition of serine proteinase activity Reduction in MET phosphorylation via inhibition of HGFA (↓ pro-HGF activation) Functioning as tumor suppressor gene Tumor-suppressive effects mediated by regulation of MMP2 expression and activity	Aberrant methylation detected in 71.4% of grade III and 74.3% of grade IV tumors. Its loss being a common event (absent in 100% of pediatric and 85.3% of adult HGG samples); increased HGFAC/SPINT2 expression ratio found in HGG; restoration suppressing cell proliferation and tumor formation in vivo.	[36,37,38,146,147]
Acute myeloid leukemia (AML)/Myelodysplastic syndrome (MDS)	downregulated	Downregulation in mesenchymal stromal cells MSCs) increasing HGF and SDF-1 secretion Enhance adhesion of malignant cells to MSCs Augmentation of HGF/c-MET signaling via increased HGFA and decreased HAI-2	Lower mRNA levels in the AML untreated group, especially in patients presenting with high white blood cell counts.	[44,148,149]
Non-small-cell lung carcinoma (NSCLC)/Lung adenocarcinoma	downregulated	Non-covalent inhibitor of plasmin Loss enhancing cell-surface plasmin activity Induction of EMT and metastasis by derepressing plasmin-mediated activation of pro-HGF and pro-TGF-β Association of downregulation with STYK1 overexpression	Downregulation predicting poor prognosis and correlating with advanced cancer stages and high tumor invasion; low expression correlating with worse OS. High expression in EGFR mutant tumors associated with shorter OS.	[150,151,152]
Renal Cell Carcinoma (RCC)	downregulated (especially in advanced stage)	Downregulation increasing hepsin/matriptase activity and enhancing ligand-dependent MET activation Loss of tumor suppressor function Restoration suppressing colony formation and cell motility	Low mRNA levels in advanced stages; downregulation in RCC presenting sarcomatoid change; low mRNA levels correlating with high hepsin mRNA level; downregulation facilitating RCC bone metastasis.	[99,153,154,155]
Ovarian cancer	downregulated; upregulated in extracellular vesicles (EVs)	Inhibition of matriptase and hepsin expression Induction of apoptosis	Low HAI-2 significantly predicting poor prognosis in terms of both DFS and OS; low expression associated with advanced stage and larger residual tumors; increased expression in ascites EVs (potential diagnostic biomarker).	[96,156,157]
Uterine leiomyosarcoma (LMS)	decreased	Anti-tumor effect mediated by downregulation of HGFA, matriptase, and hepsin Induction of apoptosis and necrosis.	Low HAI-2 levels significantly predicting poor prognosis in LMS patients.	[138]
Endometrial cancer	decreased	Overexpression suppressing proliferation, migration, and invasion Mediated by downregulation of matriptase and hepsin expression Regulation of EMT by promoting epithelial phenotype Upregulation of E-cadherin and Slug expression.	Levels negatively associated with clinicopathological parameters like lymph node metastasis and lymph vascular space involvement; low HAI-2 predicting a poor prognosis in terms of DFS and OS.	[139]
Oral squamous cell carcinoma (OSCC)	upregulated	Knockout suppressing cell proliferation and invasion Pro-invasive effects mediated by the suppression of prostasin Loss of HAI-2 resulting in prostasin upregulation	Intense immunoreactivity observed in invasive OSCC cells; high expression showing a non-significant trend toward shorter OS.	[128]
Breast cancer	upregulated	Association of high expression with promoter hypomethylation Inhibition of pro-HGF conversion mediated by HGFA, matriptase, and hepsin Cell surface translocation to inhibit active matriptase.	Correlated with tumor aggressiveness (T status, N status, and c-ErbB2 expression); high level serving as an independent negative predictive factor for poor clinical complete response to preoperative anthracycline therapy; high expression correlating significantly with shorter OS, RFS, and DMFS.	[32,56,96,110,111,112,114]
Malignant melanoma	lower in metastatic tissues	Epigenetic silencing via DNA hypermethylation Loss activating oncogenic HGF-MET signaling Ectopic expression inhibiting HGF-MET-AKT signaling	Reduction corresponding with increased DNA methylation levels; loss promoting metastatic phenotypes (cell motility and invasive growth).	[158]
Pediatric medulloblastoma (MB)	silenced/low	Epigenetic silencing via promoter CpG island hypermethylation Functioning as a candidate tumor suppressor gene	Methylation detected in 34.3% of primary MB tumors; stable re-expression in MB cells more than doubling overall survival in mouse xenograft models.	[39]
Esophageal squamous cell carcinoma	silenced/downregulated	Epigenetic inactivation via promoter hypermethylation Functioning as a tumor suppressor gene	Promoter hypermethylation detected in 52.08% of carcinoma tissues and associated with poor overall survival.	[42]
Cervical carcinoma	downregulated	Association of promotor methylation with HPV16-positive specimen Reversal of expression loss by a demethylating agent	Methylation rate (54%) higher than in normal samples; methylation status differing between grade 2 and grade 3 tumors.	[43]

OS: overall survival, RFS: recurrence-free survival, DMFS: distant metastasis-free survival.

## 6. Future Directions

### 6.1. Basic Research in Physiological Functions

Although the target serine proteases of HAI-1 and HAI-2 have been identified in several organs, many other tissues and specialized cells still express HAI-1 and HAI-2 without known corresponding target proteases or downstream substrates. Elucidating these unknown targets and substrates will reveal novel and important physiological functions of HAI-1 and HAI-2 in these sites. A notable example is the recent discovery of HAI-1′s role in regulating insulin synthesis in pancreatic β-cells [103]. The next critical questions to address are whether HAI-1 and HAI-2 exhibit distinct, non-overlapping functions in these tissues or specialized cell types, what regulatory cascades are associated with their respective target substrates, and how these cascades ultimately integrate external stimuli to orchestrate multilayered responses in pericellular proteolysis. High-throughput degradomics may be useful to map these inhibitor-protease networks across different tissues and cell types. In addition, future investigations into HAI-1 and HAI-2 should extend beyond their established roles as mere proteolytic inhibitors to elucidate their complex non-canonical functions. For example, the unique MANEC domain of HAI-1 likely mediates specific protein–protein interactions [159], while HAI-2 has been shown to possess critical intracellular chaperone capabilities distinct from its inhibitory role [31,62]. Uncovering these non-canonical or proteolysis-independent functions would significantly expand our understanding of the physiological roles of HAI-1 and HAI-2.

### 6.2. Basic Research in Cancer Biology

A critical research priority is deciphering the double-edged sword paradox in oncology: although HAI-1 and HAI-2 function as tumor suppressors in most cancers, they are paradoxically upregulated in breast and hepatocellular carcinomas to promote cancer cell survival [110,141]. Future research should aim to define the specific microenvironmental cues, such as hypoxia, *HER2* amplification, or serine proteases or modifiers that are tissue- or tumor-type-specific, which convert these inhibitors from suppressors into facilitators for cancer cell survival [56].

### 6.3. Therapeutic Opportunities and Targeting Strategies for Cancer Treatment

Dysregulation of the inhibitory axes formed by HAI proteins and their target TTSPs is increasingly recognized as a driver of tumor invasion and metastasis, thereby presenting multiple therapeutic opportunities.

One of the most direct strategies is functional restoration of HAI-1 or HAI-2 activity. Experimental re-expression or overexpression of HAI-1 or HAI-2 using viral or vector-based overexpression methods has been shown to suppress proliferation, invasion, and metastasis in multiple epithelial cancer models, including bladder [160], liver [161], and lung cancers [150]. Gene therapy-like approaches remain technically challenging and require more effective, controllable, and safer delivery methods. In addition, recombinant proteins or mRNA therapies with lipid nanoparticles may serve as alternative approaches, offering the advantage of avoiding insertional mutagenesis. Recombinant HAI-2 proteins containing the extracellular region of HAI-2 have been shown to inhibit cell migration and invasion in models of breast [112], prostate [27], and lung cancers [150]. Moreover, it has been shown that the KD1 domain, but not the KD2 domain, exerts anti-tumor activity. Indeed, recombinant KD1 has been shown to suppress invasion and metastasis of PCa [162], as well as metastasis of pancreatic cancer in animal models [159]. Developing engineered HAI-1 and HAI-2 mimetics based on the KD1 domain and applying them in cancers where low HAI expression predicts poor survival could represent a novel antitumor strategy. Future efforts should focus on improving the stability and tumor penetration of recombinant KD1 or KD1 mimetics, while also addressing challenges such as immunogenicity and off-target inhibition of multiple serine proteases, which may lead to toxicity or side effects.

Another feasible strategy is the epigenetic reactivation of silenced *SPINT1* or *SPINT2* genes, as they are frequently downregulated in several cancers through promoter hypermethylation or other epigenetic mechanisms. Reactivation using demethylating agents such as 5-aza-2′-deoxycytidine has been shown to restore *SPINT2* expression, thereby inhibiting migration and invasion of liver cancer cells in vitro and suppressing tumorigenicity in vivo [161]. This highlights the therapeutic potential of the approach that targets an upstream regulatory defect rather than individual downstream signaling events. In this regard, other epigenetic-modifying agents or methods may be explored, such as additional demethylating agents like guadecitabine [163], histone deacetylase inhibitors [164], and CRISPR activation systems [165], all of which have been shown to successfully reactivate certain tumor suppressor genes. Furthermore, given that HAI dysregulation mediates resistance to EGFR and c-MET inhibitors in colorectal and lung cancers, future preclinical trials may leverage HAI re-expression to guide combinatorial therapies aimed at overcoming drug resistance [135,136].

Alternative strategies include direct inhibition of target proteases such as matriptase and HGFA, using synthetic small molecules [166,167,168], engineered peptides [169], or antibodies [170]. Targeting downstream pathways such as the HGF/MET axis and PAR-2 signaling offers another approach, with combination therapies showing strong promise. A third strategy involves intracellular targeting, as engineered HAI variants capable of modulating non–surface-localized protease activity could change the stability of TTSPs [62] and expand the therapeutic scope beyond extracellular proteolysis.

Given the link between HAI-1 and M2 macrophage polarization in NSCLC [127,136], future work should explore combining HAI-targeted therapies with anti-PD-1/PD-L1 immunotherapies. Restoring HAI-1 function might re-prime the TME to be more receptive to immune checkpoint inhibitors by shifting the macrophage balance toward an anti-tumor M1 phenotype. On the other hand, it has been shown that targeting serine protease activity in the tumor microenvironment using a synthetic small-molecule inhibitor can overcame fibroblast-mediated resistance to MET inhibition in NSCLC cells by preventing fibroblast-mediated reactivation of AKT and ERK signaling [171]. There still is a vast, relatively less explored territory regarding how these inhibitors modulate the tumor TME and immune evasion in human tumors other than NSCLC, where HAI-1 and HAI-2 have been shown involved. For example, future research may investigate whether the loss of HAI-1/2 affects the recruitment of myeloid-derived suppressor cells or the exhaustion of T cells in common human cancers mentioned in Table 1 and Table 2.

### 6.4. Other Diseases and Clinical Translation

Beyond oncology, the roles of HAIs in development suggest that modulating the HAI-protease axis offers novel interventions for genetic and metabolic diseases. Since the lethal phenotypes of *Spint1* or *2* deficiency can be rescued by ablating their target proteases, synthetic small-molecule inhibitors of matriptase or prostasin could functionally compensate for HAI insufficiency in disorders like SCSD or congenital ichthyosis [64]. This approach could also be extended to other common applications in regenerative medicine, such as promoting epithelial repair following injury or chronic inflammation. In the context of metabolic health, the recent identification of the *Spint1*-hepsin-GLP1R axis highlights new therapeutic targets for diabetes. Specific hepsin inhibitors could mimic the protective effect of HAI-1 on insulin receptors to improve glucose tolerance [103]. Additionally, given HAI-2′s ability to inhibit the activation of influenza and metapneumoviruses [15], developing HAI-based antiviral coatings or inhalants could represent a promising future approach for preventing viral entry in the airway.

## 7. Conclusions

This review underscores the critical structural features and regulatory functions of HAI-1 and HAI-2 related to TTSPs, highlights their essential roles in maintaining tissue homeostasis, and discusses how their downregulation or hijacking by tumors contributes to cancer progression. Despite structural similarities and certain overlapped functions, HAI-1 and HAI-2 exhibit divergent subcellular localizations: HAI-1 is primarily located on the cell surface, where it inhibits active proteases, whereas HAI-2 is predominantly cytoplasmic with some apical membrane presence and contributes to the maturation and trafficking of TTSPs. They also exhibit distinct, non-redundant physiological roles: HAI-1 functions as a primary epithelial barrier guardian, essential for placental labyrinth formation and the prevention of skin ichthyosis, while HAI-2 is involved in neural tube closure, regulation of iron homeostasis, and protection against thoracic aortic dissection. In cancers, HAI-1 and HAI-2 predominantly act as tumor suppressors by inhibiting HGF/MET and matriptase signaling, and their genes are therefore frequently silenced through hypermethylation. However, in certain cancers, they are paradoxically upregulated. These context-dependent and opposing roles may reflect the influence of distinct tissue- or tumor-specific protease signatures. Future research should further define the unknown target proteases and non-canonical functions of HAI-1 and HAI-2, especially in tissues where their physiological roles remain unclear. Understanding how these proteins shift from tumor suppressors to promoters of cancer progression in a context-dependent manner may uncover therapeutic opportunities, including epigenetic reactivation, mimetic design, and immunotherapy combinations. Specifically, frequent SPINT1/2 silencing via promoter hypermethylation offers a target for epigenetic therapy. DNMT1 inhibitors restore SPINT2 expression and suppress HGF/MET signaling in preclinical models, highlighting epigenetic therapy as one of the most promising therapeutic strategies in cancer. Finally, a number of TTSPs and related soluble serine proteases still lack well-defined functions, particularly in tissues or cell types where they are enriched but remain poorly characterized. Therefore, although much has been learned about the functions of HAI-1 and HAI-2, this field will undoubtedly continue to expand. Moreover, the development and clinical translation of HAI-based therapeutic strategies represent an exciting and evolving frontier.

## Data Availability

No new data were created or analyzed in this study. Data sharing is not applicable to this article.

## Abbreviations

The following abbreviations are used in this manuscript (in order of first mention):

TTSPs	Type II transmembrane serine proteases
TMPRSS2	Transmembrane protease, serine 2
HAI-1	Hepatocyte growth factor activator inhibitor-1
SPINT1	Serine peptidase inhibitor, Kunitz Type 1
HAI-2	Hepatocyte growth factor activator inhibitor-2
SPINT2	Serine peptidase inhibitor, Kunitz Type 2
HGF	Hepatocyte growth factor
HGFA	Hepatocyte growth factor activator
MET	Mesenchymal–epithelial transition factor
KD1	Kunitz domain 1
KD2	Kunitz domain 2
MANEC	Motif at the N-terminus with eight cysteines
PKD	Polycystic kidney disease
SCSD	Syndromic congenital sodium diarrhea
ER	Endoplasmic reticulum
HCC	Hepatocellular carcinoma
mRNA	Messenger ribonucleic acid
DNMT1	DNA methyltransferase 1
PCa	Prostate cancer
GRHL2	Grainyhead-like-2
SMG	Submandibular salivary gland
MMP	Matrix metalloproteinase
uPA	Urokinase-type plasminogen activator
sHAI-1	Soluble HAI-1
HIF	Hypoxia-inducible factor (included HIF-1α/2α)
maHAI-1	Membrane-anchored recombinant HAI-1
HAT	Human airway trypsin-like protease
EpCAM	Epithelial cell adhesion molecule
PAR-2	Protease-activated receptor-2
TROP2	Trophoblast cell surface antigen 2
SNP	Single-nucleotide polymorphism
GI	Gastrointestinal
SF	Scatter factor
E	Embryonic day
KD	Knockdown
BCT	Basal chorionic trophoblast
MT2	Matriptase-2
ENaC	Epithelial sodium channel
SOX9	SRY-box transcription factor 9
OA	Osteoarthritic
GLP1R	Glucagon-like peptide 1 receptor
NPCs	Neural progenitor cells
BMP	Bone morphogenetic protein
CP	Choroid plexus
CPE	Choroid plexus epithelial
SIP1	Smad-interacting protein 1
AD	Alzheimer’s disease
TAD	Thoracic aortic dissection
SMCs	Smooth muscle cells
PDGF-BB	Platelet-derived growth factor BB
TME	Tumor microenvironment
OSCC	Oral squamous cell carcinoma
CAF	Cancer-associated fibroblast
AML	Acute myeloid leukemia
MSCs	Mesenchymal stromal cells
SDF-1	Stromal cell-derived factor-1
EMT	Epithelial–mesenchymal transition
NSCLC	Non-small-cell lung carcinoma
RCC	Renal cell carcinoma
LMS	Leiomyosarcoma
OS	Overall survival
RFS	Recurrence-free survival
DMFS	Distant metastasis-free survival
HGG	High-grade glioma
MB	Medulloblastoma
